# Pleiotropic Effects of Heparin and its Monitoring in the Clinical Practice

**DOI:** 10.1055/s-0044-1786990

**Published:** 2024-05-29

**Authors:** Deepa J. Arachchillage, Steve Kitchen

**Affiliations:** 1 Centre for Haematology, Department of Immunology and Inflammation, Imperial College London, London, United Kingdom; 2 Department of Haematology, Imperial College Healthcare NHS Trust, London, United Kingdom; 3 Department of Coagulation, Royal Hallamshire Hospital, Sheffield, United Kingdom

**Keywords:** unfractionated heparin, activated partial thromboplastin time, heparin anti-Xa, activated clotting time, pleotropic effects

## Abstract

Unfractionated heparin (UFH) was uncovered in 1916, has been used as an anticoagulant since 1935, and has been listed in the World Health Organization's Model List of Essential Medicines. Despite the availability of many other anticoagulants, the use of heparin (either low molecular weight heparin [LMWH] or UFH) is still substantial. Heparin has pleotropic effects including anticoagulant and several nonanticoagulant properties such as antiproliferative, anti-inflammatory activity, and anticomplement effects. Although UFH has been widely replaced by LMWH, UFH is still the preferred anticoagulant of choice for patients undergoing cardiopulmonary bypass surgery, extracorporeal membrane oxygenation, and patients with high-risk mechanical cardiac valves requiring temporary bridging with a parenteral anticoagulant. UFH is a highly negatively charged molecule and binds many positively charged molecules, hence has unpredictable pharmacokinetics, and variable anticoagulant effect on an individual patient basis. Therefore, anticoagulant effects of UFH may not be proportional to the dose of UFH given to any individual patient. In this review, we discuss the anticoagulant and nonanticoagulant activities of UFH, differences between UFH and LMWH, when to use UFH, different methods of monitoring the anticoagulant effects of UFH (including activated partial thromboplastin time, heparin anti-Xa activity level, and activated clotting time), while discussing pros and cons related to each method and comparison of clinical outcomes in patients treated with UFH monitored with different methods based on available evidence.

Heparan sulfate proteoglycans are found almost universally on the cell surface and in the extracellular matrix of mammalian cells. 1 Heparan sulfate proteoglycans have a wide range of biological functions such as regulation of growth, cell adhesion, 2 and anticoagulant properties, mainly through enhancing the inhibitory activity of antithrombin (AT). 3 Heparin is related to heparan sulfate and is synthesized in mast cells. Under physiological conditions, heparin is not found within the vascular endothelium; however, upon vascular injury, heparin is released into the site of injury from the secretory granules of basophils and mast cells. 4 At the site of vascular injury, heparin helps maintain normal blood flow by balancing the active anticoagulant and procoagulant processes. 4 Pharmaceutical grade heparin is derived from animal tissue, mainly from porcine gut mucosa. 5 Unfractionated heparin (UFH) was uncovered in 1916, 6 has been used as an anticoagulant since 1935, 7 and has been listed in the World Health Organization's Model List of Essential Medicines. 8 Despite the availability of many of other anticoagulants, demand for heparin has not reduced and in fact, during the coronavirus disease 2019 (COVID-19) pandemic, the use of heparin increased significantly. 9 Heparin is a mixture of sulfated polysaccharide with an average molecular weight of 15,000 Da, but varies from 3,000 to 30,000 Da.

Although UFH has been widely replaced by low molecular weight heparin (LMWH), UFH is still the preferred anticoagulant of choice for patients undergoing cardiopulmonary bypass (CPB) surgery, extracorporeal membrane oxygenation (ECMO) at many centers, especially in the United Kingdom, 10 and patients with high-risk mechanical cardiac valves needing temporary bridging with a parenteral anticoagulant. 11 Due to the short half-life of UFH, excretion is not dependent on renal function, with anticoagulant action reversed readily with protamine sulfate.

UFH is a highly negatively charged molecule, which helps its binding to positively charged surfaces and molecules including endothelial cells, macrophages, acute phase proteins, platelet factor 4 (PF4) released from activated platelets, and high von Willebrand factor (VWF) molecular weight multimers, which are increased in critically ill patients in whom UFH is commonly used in clinical practice. 10 Due to this nonspecific binding, UFH has unpredictable pharmacokinetics, and anticoagulant effects. 12 Therefore, the anticoagulant effect of UFH may not be proportional to the dose of UFH given to any individual patient. Furthermore, the unpredictability and variability of the activated partial thromboplastin time (aPTT), the most commonly used laboratory method used for monitoring the anticoagulant effects of UFH, 13 14 15 make it an unreliable measure or UFH and thus challenge its use in clinical practice. LMWH represents a fractionated form of heparin with an average molecular weight of 5,000 Da. Similar to UFH, LMWHs also exert their anticoagulant activity by catalyzing the inhibition of coagulation factors by AT. This interaction with AT is mediated by a unique pentasaccharide sequence which requires at least 18 saccharide units to enhance neutralization of thrombin.

LMWH has more predictable anticoagulant effects than UFH and monitoring is required only in certain situations, such as renal failure, bleeding, extreme body weight or during pregnancy, or when used in patients with high-risk mechanical heart valves. 12 16 17 Monitoring of LMWH should be done using an anti-Xa assay calibrated with material traceable to the International Standard for LMWH, with results reported in IU/mL, 18 since clot-based tests including aPTT are unsuitable for LMWH monitoring. 18 Osteoporosis and heparin-induced thrombocytopenia (HIT) also are less common complications of LMWH compared to UFH. 19

In this review, we discuss the anticoagulant and nonanticoagulant activities of UFH, differences between UFH versus LMWH, when to use UFH, different methods of monitoring the anticoagulant effects of UFH, also discussing pros and cons related to each method, and comparison of clinical outcomes in patients treated with UFH monitored with different methods based on available evidence.

## Anticoagulant Activity of Heparin

Around one-third of administered UFH binds to AT and this accounts for the majority of its anticoagulant effects when heparin is given at therapeutic concentrations. 20 Heparin enhances inhibitory activity of AT against several serine proteases involved in the coagulation process, most importantly thrombin (factor IIa), factor IXa (FIXa), and factor Xa (FXa). 21 The binding of heparin to AT induces conformational changes in AT, leading to both the removal of the reactive loop and exposure of exosites on the surface of AT, which bind directly to the enzyme target; a template mechanism also exists in which both inhibitor and enzyme bind to the same heparin molecule. 22 The degree of inhibition of thrombin and FXa depends on the molecular weight of heparin. Once activated, AT can inhibit FXa and FIXa several hundred fold faster than the native serpin. 2 23 Unlike rapid inhibition of FXa and FIXa, the conformational activation of AT does not provide an important contribution to inhibition of thrombin. 24 The increased inhibition of thrombin by AT requires a heparin with a higher molecular weight, with sufficient chain length in order to link both thrombin and AT in a ternary complex. This explains why UFH is able to inhibit both thrombin and FXa in a 1:1 ratio, while LMWH is a better target for FXa than for FIIa (FIIa:FXa inhibition ratios ranging from 1:2 to 1:4). 3

In addition to inhibition of thrombin and FXa, the heparin–AT complex inactivates several other coagulation enzymes including FVIIa, FXIa, and FXIIa. 25 Furthermore, heparin can act through other serine protease inhibitors such as heparin cofactor II, and heparin induces release of tissue factor pathway inhibitor and inhibits platelet and VWF binding. 26

## Nonanticoagulant Activities of Heparin

In addition to its well-known anticoagulant effects described above, there is considerable evidence that heparin has several nonanticoagulant effects, including anti-inflammatory activity, both experimentally and clinically, and anticomplement effects. 27 Notably, in many instances, these actions of heparin are independent of anticoagulant activity, raising the possibility of developing novel drugs based on heparin that retain the anti-inflammatory activity. Heparin exerts anti-inflammatory activities via a variety of mechanisms, including inhibition of adhesion molecules, and the inhibition of heparinase, all involved in leukocyte recruitment into tissues and neutralization of cationic mediators. 2

Possible anti-inflammatory effects of heparin have been discussed in detail by others. 28 29 Binding of heparin to different mediators implicated in the immune response (including cytokines, selectins, 30 angiogenic factors, integrins, PF4, 31 chemokines, 32 acute phase response proteins, 33 and complement components 34 ) may contribute to its anti-inflammatory effects. 35 It is suggested that heparin can neutralize cytokines at the site of inflammation. Several studies have demonstrated that patients treated with heparin had reduced levels of cytokines such as tumor necrosis factor-α, 36 interleukin-6, interleukin-8, and C-reactive protein. 37 38 Through its binding to P-selectin, heparin could inhibit adhesion of leukocytes and neutrophils to endothelial cells and thereby prevent the release of oxygen radicals and proteolytic enzymes. 30

It is postulated that the benefit of heparin in obstetric antiphospholipid syndrome (APS) is due to inhibition of apoptosis, inflammation, and complement activation rather than anticoagulant effects. 39 These effects of heparin are particularly relevant for prevention of early recurrent miscarriages in which the placenta is not well-formed. Therefore, it is less likely that prevention of placental thrombosis is the main mechanism that helps heparin to prevent early miscarriage. Furthermore, possible contribution of complement activation may be of more relevance beyond the early pregnancy complications, and it has been assumed that the nonanticoagulant effects of heparins on inflammatory processes, vascular function, or placental pathology may also play a role in prevention of preeclampsia, a late pregnancy complication strongly associated with APS. 40 In a mouse model of APS, Girardi et al elegantly demonstrated that treatment with heparin (UFH or LMWH) inhibited complement activation *in vivo* and *in vitro* and protected mice from pregnancy complications induced by antiphospholipid antibodies. 39 However, fondaparinux (a synthetic anticoagulant retaining the essential pentasaccharide structure of LMWH) or hirudin (a naturally occurring anticoagulant peptide derived from leeches) did not inhibit the generation of complement split products or were not able to prevent pregnancy loss miscarriage induced by antiphospholipid antibodies. 39

Additional nonanticoagulant effects of heparins include binding of growth factors to cell surface, reducing bone osteoblast activity with more propensity to cause osteoporosis, antiproliferative effects including inhibition of smooth muscle cell, and endothelial cell proliferation. 22 23 However, meta-analyses of patients treated with heparin showed conflicting results. Earlier meta-analysis showed cancer patients treated with LMWH had significantly higher survival than those with no history of venous thromboembolism (VTE), although an excess of all bleeding events and major bleeding were seen in those treated with LMWH. 24 A subsequent meta-analysis in 2014 showed no benefit in survival of cancer patients treated with LMWH. 41 This contrasts with a 2022 meta-analysis which included 2,617 patients from 15 randomized controlled trials evaluating the efficacy of UFH in patients with sepsis 42 which concluded that UFH could reduce 28-day mortality (relative risk [RR]: 0.82; 95% CI: 0.72–0.94) compared to patients who did not receive UFH. 42

## Unfractionated Heparin versus Low Molecular Weight Heparin

UFH has been largely replaced by LMWH due to more reliable dose-responsive anticoagulant effect, no need for regular monitoring of the anticoagulant effect, longer half-life, subcutaneous injection, and lower incidence of HIT and osteopenia. 10 43 44 However, UFH is the preferred anticoagulant of choice for patients undergoing cardiac surgery, patients supported by ECMO, and critically unwell patients in the intensive care units requiring anticoagulation due to shorter half-life, independence from renal function, and reversibility by protamine sulfate. 10 45 46

Several large studies demonstrated that there were no significant differences in the safety and efficacy of LMWH compared to UFH in treatment of venous thromboembolism. 47 48 Two meta-analyses reported that LMWHs are safer and more effective than UFH. 49 50 In terms of side effects of heparin, although it is rare, HIT remains the most serious concern. Incidence of HIT is around five times higher under treatment with UFH compared to LWMH. In the meta-analysis by Martel et al, which included largely orthopaedic patients, the incidence of HIT with LMWH was 0.2 in comparison to 2.6% with UFH. 51 A Cochrane review found a similar risk following surgery, where incidence of HIT with UFH was 2.2 compared to 0.5% in patients receiving LMWH. 52 The main differences between UFH and LMWH are summarized in Table 1 .

**Table 1:** Differences between unfractionated heparin and low molecular weight heparin

Characteristic	UFH	LMWH
Average molecular weight	15 kDa	4.5 kDa
Anticoagulant activity	Inhibits FIIa and FXa in a 1:1 ratio	FXa is a better target than FIIa (FIIa:FXa inhibition ratios ranging from 1:2 to 1:4) 3
Mode of administration	Continuous infusion or subcutaneous injections	Subcutaneous injections
Excretion	Saturable mechanism represents clearance by the reticuloendothelial system and endothelial cells, or nonsaturable renal excretion may be important when very high doses are used	Nonsaturable renal excretion
Half life	Dose-dependent, and ranges from 0.5 to 2 hours and increases with increasing doses	3 to 4 hours
Reversal	Reversal with protamine sulfate is possible	Protamine sulfate reversal possible but incomplete
Monitoring	When given by intravenous infusion, monitoring is essential due to high nonspecific binding	Monitoring is required only in specific situations such as renal failure, extreme body weight, during pregnancy, or patients with active bleeding
Methods of monitoring	aPTT or heparin anti-Xa level	Heparin anti-Xa level
Risk of osteoporosis	Higher	Lower
Risk of heparin induced thrombocytopenia	Higher (approximately five times higher than LMWH) 51 52	Lower

Abbreviations: aPTT, activated partial thromboplastin time; FXa, factor Xa; LMWH, low molecular weight heparin; UFH, unfractionated heparin.

## Measuring Unfractionated Heparin Activity and Therapeutic Ranges

The two most widely used laboratory tests for monitoring for UFH in clinical practice are aPTT and heparin anti-Xa assay. However, neither of these assays has the ability to capture all of the antithrombotic effects of UFH; in addition, both approaches have technical limitations. Furthermore, the clinical evidence to support the widely used therapeutic ranges for aPTT or anti-Xa for UFH is rather weak at present. Both will be discussed below and as shown in Table 2 .

**Table 2:** Activated partial thromboplastin time versus heparin anti-Xa in monitoring unfractionated heparin

aPTT	Heparin anti-Xa
Measures time taken (in seconds) to clot formation following activation with calcium and intrinsic coagulation pathway activator such as silica or ellagic acid	Determines anticoagulant activity by measuring the ability of the heparin–antithrombin complex to inhibit factor Xa Any drug with anti-Xa activity will yield a result (UFH, LMWH, fondaparinux, anti-Xa DOACs), accurate drug quantification requires calibration with the correct drug
Variation among the sensitivities of different aPTT reagents and susceptibility to factors that do not reflect intrinsic heparin activity (nonspecific for heparin)	Since the anti-Xa measures the inhibition of a single enzyme, it is a more direct measure of UFH activity than the aPTT
aPTT is significantly affected by variation in coagulation factors (i.e., FVIII and fibrinogen especially when raised during infection/inflammation, or FXII deficiency) and LA	Minimal interference from the presence of biologic factors, such as LA and variation in coagulation factors
Readily available, ease-of-automation, and relatively low cost per test	Relatively high cost per test, but per person overall cost may be same or lower when compared to aPTT, due to overall lower number of tests and therapeutic range is achieved quicker; not as widely available
Affected by multiple activities of UFH, including anti-Xa and anti-IIa	Only considers anti-Xa activity, not anti-IIa activity

Abbreviations: aPTT, activated partial thromboplastin time; DOAC, direct oral anticoagulant; LA, lupus anticoagulant; UFH, unfractionated heparin.

### Preanalytics for Activated Partial Thromboplastin Time or Anti-Xa in Samples Containing Unfractionated Heparin

Appropriate collection and processing of the blood samples to prepare platelet-poor plasma to assess aPTT or heparin anti-Xa assay are vital. Platelet activation occurs over time in citrated blood, after blood is added to the collection tube, leading to release of PF4. If UFH is present in the sample, there is time-dependent neutralization of that heparin, which in turn may lead to aPTT shortening. 53 Citrate, theophylline, adenosine, and dipyridamole (CTAD) can also be used as anticoagulant when collecting samples for aPTT, although these may generate changes in reference and heparin therapeutic ranges. The citrate in CTAD chelates the calcium and prevents in vitro platelet activation. Samples from patients receiving UFH collected into CTAD are stable for at least 4 hours, 53 54 whereas the International Council for Standardization in Haematology (ICSH) have recommended that citrated blood samples containing UFH received in the laboratory for aPTT should be centrifuged within 1 hour of collection and the analysis completed within 4 hours of collection. 55

Some studies have indicated that centrifugation can be delayed for up to 4 hours prior to heparin anti-Xa assay since there was only minor loss of anti-Xa activity and little clinically relevant impact on management decisions when tested using an assay containing dextran sulfate (DS), which causes disassociation of heparin–PF4 complexes. 56 Another study demonstrated that there was no loss of heparin anti-Xa in citrated blood for assays with or without DS included in reagents up to 6 hours in whole blood or plasma. 54 Some collection tubes are designed so that there is large residual air space even after the correct volume of blood is added to anticoagulant. This large air space in the tube after addition of blood to anticoagulant in the correct ratio may affect test results. This is due to the additional available space for sample mixing during transport and the higher blood collection tube inner surface-to-blood volume ratio, which can lead to platelet activation, release of PF4, and neutralization of a proportion of UFH with loss of heparin anti-Xa activity and shortening of aPTT if citrate is used as anticoagulant 57 and use of CTAD can overcome this issue. 58 ICSH have recently recommended that blood samples collected into trisodium citrate for monitoring UFH therapy should not have a large air space after addition of blood sample to the tube is completed 59 and that if citrate is used for blood samples for determination of aPTT, the tubes should contain 105 to 109 mmol/L (3.1–3.2%) of trisodium citrate. 59

### Activated Partial Thromboplastin Time for Monitoring Unfractionated Heparin

A major problem related to use of the aPTT for monitoring UFH is the lack of specificity for UFH effect. Due to various confounding factors, aPTT can be either disproportionately prolonged or shorter despite having expected therapeutic heparin anti-Xa level. Presence of excess anticoagulant (i.e., sodium citrate) in the blood collection tube, including underfilled tubes, raised hematocrit, presence of lupus anticoagulant (LA) or coagulation factor deficiency can all contribute to prolonged aPTT independent of heparin present in the sample. 60 There are also occasions when the aPTT is below the therapeutic range when there is a therapeutic level of UFH in the sample. This frequently occurs when FVIII and/or fibrinogen levels are markedly elevated in patients receiving UFH due to acute phase response. 10 Indeed FVIII is often elevated in patients with acute thromboembolic events via other mechanisms 61 and also during pregnancy. 62 The first study that established a therapeutic range for UFH using aPTT was a small (234 patients) prospective study in 1972, where two-thirds of cases had VTE and one-third had arterial thrombosis. 63 The authors reported a low risk of recurrent thrombosis when a target aPTT ratio of 1.5 to 2.5 times compared to control aPTT ratio, but the number of events was low. 63 The evidence to support a ratio of 2.5 as the upper limit of therapeutic range was particularly weak. 63 We now know that there are very large differences in the responsiveness of aPTT reagents to the effect of UFH, and many different commercial reagents are available. 64 65 The aPTT reagent used by Basu et al 63 has long since disappeared from usage, so it is not possible to directly compare results with that method to reagents in use today. On the other hand, the same group later reported that the aPTT range of 1.5 to 2.5 by the method they used corresponds to 0.2 to 0.4 U/mL heparin when measured using a protamine titration assay or a range of 0.3 to 0.7 IU/mL by an anti-Xa assay. 66 There are studies which demonstrate the ability to compensate for aPTT reagent variability by comparing results against a heparin assay either by protamine titration 64 65 or by anti-Xa assay. 67

If aPTT is used for monitoring of UFH, for example, if an anti-Xa assay is not available, the range of the therapeutic aPTT equivalent to heparin anti-Xa of 0.3 to 0.7 IU/mL should be established locally by comparison of aPTT and heparin anti-Xa activities determined on samples from patients receiving UFH therapy. 10 68 The regression relationship is used to calculate the aPTT or aPTT ratio range equivalent to heparin anti-Xa 0.3 to 0.7 IU/mL heparin. In such comparisons, the correlation between heparin anti-Xa and aPTT is generally poor because of the lack of specificity of aPTT for heparin effect. Some authors have used several hundred samples in such a comparison for this reason 69 but this would be increasingly difficult in centers where there has been a decline in use of UFH and replacement by alternative anticoagulants. A minimum of 20 was recommended by some experts, 70 whereas other groups used 30 to 60 samples in similar comparisons. 10 64 65 Comparison of aPTT and heparin anti-Xa to establish a therapeutic range by aPTT should not be done using samples constructed by heparin-spiked (“in vitro”) plasma, since this does not generate samples which behave in the same way as samples collected (“ex vivo”) from patients generating misleading information on heparin sensitivity. 71

It is not known to what extent use of a locally determined aPTT therapeutic range as described above improves interlaboratory agreement of UFH monitoring in terms of whether a patient is considered to be subtherapeutic, therapeutic, or supratherapeutic. One study compared patient aPTTs in four accredited laboratories using three different aPTT reagents/four different analyzers by comparing an aPTT therapeutic range calibrated against heparin anti-Xa to uncalibrated aPTT ranges. 72 This study found that there was a failure to improve interlaboratory consensus on whether a patient's result indicated subtherapeutic, therapeutic, or supratherapeutic control when calibrated ranges were used in 44 UFH patients compared to use of uncalibrated aPTT ranges. 72 Others have adopted composite therapeutic ranges by combining data from different sites in a network using the same aPTT reagent and analyzers from the same family. 73

If a patient has a prolonged aPTT before commencement of UFH therapy, then that aPTT method cannot be safely used to monitor UFH therapy, as their aPTT may overestimate the anticoagulant of UFH, leading to decreased UFH dosing and increased risk of thrombosis. If the preheparin therapy aPTT is below the lower limit of the normal reference range, then UFH therapy should be initiated with caution and if the baseline aPTT is markedly shorter, once again aPTT may not be safe to use for monitoring as the aPTT significantly underestimates anticoagulant effect of UFH leading to possible increased risk of bleeding. These problems with aPTT suggest that there are advantages to using heparin anti-Xa assay for monitoring UFH therapy.

### Heparin Anti-Xa for Monitoring Unfractionated Heparin

A range of 0.3 to 0.7 IU/mL by anti-Xa assay is the widely accepted therapeutic range for UFH therapy 14 18 for treatment doses. However, the evidence to support this comes from a single, small study. 67 The study assessed patients who required larger than average doses of heparin to achieve the therapeutic target range for heparin anti-Xa. Patients were randomized to be monitored by aPTT or by anti-Xa assay and for both groups, the therapeutic range used was equivalent to 0.2 to 0.4 U/mL by protamine titration. 67 The heparin anti-Xa range was 0.35 to 0.67 IU/mL, which was subsequently rounded to 0.3 to 0.7 IU/mL in later guidance/guidelines referencing this study. 66 It is important to note that the heparin anti-Xa assay reagents (Stachrom Heparin, Diagnostica Stago, France) used includes AT in the reagents and does not contain DS 74 (this is discussed in more detail below). The group monitored by aPTT received higher mean daily doses of UFH than the group monitored by heparin anti-Xa. 67 The patients were followed for 12 weeks after initiation of heparin therapy for the assessment of recurrent VTE. There were no significant differences in the recurrent VTE events between patients monitored by aPTT versus heparin anti-Xa (3/65 recurrent VTE events in anti-Xa group vs. 4/66 recurrent VTE events in aPTT group). 67 There were four bleeding events in the aPTT group and one in the group monitored by anti-Xa. 67 The authors concluded that the lower dosages of UFH could be used if anti-Xa monitoring is used. 67 This was the first study to the best of our knowledge that described advantages for anti-Xa assay over aPTT.

DS is present in reagents used for some versions of heparin anti-Xa assay but is absent from others. The stated purpose of adding DS to heparin anti-Xa assay is to release UFH bound to plasma proteins. 75 Such binding of UFH to plasma proteins may occur in vivo or in vitro. One such protein is PF4, which is well-known to be released from platelets in vitro after sample collection, as described above. In this case UFH is bound in vitro and its activity/effect is lost, leading to lower heparin activity available for measurement in the collection tube than that available for therapeutic effect in the patient. 75 It might be considered an advantage to release heparin from complexes with proteins if the binding has occurred in vitro so that the assay of heparin effect better reflects the available heparin activity in vivo. On the other hand, if UFH is bound in vivo, and is unavailable to exert its anticoagulant effects in such complexes, then use of an assay without DS, where heparin is not released from complexes formed in vivo, may better reflect anticoagulant potential in the patient. Indeed it has been reported that anti-Xa determined using reagents containing DS can give higher values than anti-Xa assays lacking DS 75 76 and in some settings this may be a consequence of release of heparin from heparin/protein complexes formed in vivo. This has been reported in a study of cardiac surgery patients, when samples were collected after heparin reversal by protamine. 77 However, there is no consensus on whether DS should be present in heparin anti-Xa assay reagents and there are multiple commercial kits available for heparin anti-Xa with or without DS. A recent reevaluation of the impact of DS concluded that high concentrations of DS lead to higher than predicted FXa inhibition when UFH is added to plasma. 78 Based on their findings the authors suggested to use dextran-free anti-Xa assays, provided that the blood collection is performed carefully and with a recommendation to discard the first few milliliters of blood collected following venepuncture before collecting the sample used for anti-Xa assay to limit the amount of PF4 produced artifactually. 78

Despite presence or absence of DS affecting heparin anti-Xa assay results, 76 interassay agreement for different heparin anti-Xa assays is much better than that seen for different aPTT methods in samples from patients on UFH. For example, the between-center coefficient of variation (CV) at therapeutic levels of UFH was approximately 10% for anti-Xa compared to 15 to 25% of CVs seen in proficiency testing surveys for aPTT determined with multiple reagents. 76

If aPTT is used there is a requirement to validate the therapeutic range after a change in lot number of aPTT reagent. 70 This is not necessary for heparin anti-Xa, where the target range in IU/mL remains the same. Another advantage favoring anti-Xa over aPTT is that use of anti-Xa monitoring of UFH achieving therapeutic values faster and with fewer dose adjustments compared to use of aPTT. 79 80

Although difficult to perform, the best studies are those which consider clinical outcomes comparing heparin anti-Xa versus aPTT. There is a large (20,000 cases) retrospective cohort study which found that patients monitored by anti-Xa were less likely to have a transfusion while hospitalized than the patients who were monitored by aPTT (after controlling for age, gender, other risk factors, and invasive procedures). 81 Thus, there are a number of potential and published advantages of anti-Xa over aPTT for monitoring UFH. A meta-analysis of 10 studies which included data from more than 6,500 cases found that use of anti-Xa compared to aPTT was not associated with increased risk of thrombotic events (RR 0.99; 95% CI 0.76–1.30) or bleeding (RR 1.03; 95% CI 0.8–1.22). 82 There were no differences in mortality in individual studies analyzed, although the authors considered that the data were not suitable for pooled analysis by their criteria. 82 Heparin requires AT to inhibit coagulation enzymes and drug concentration may be underestimated when AT falls below 50 IU/dL 75 ; therefore, some anti-Xa assay kits contain AT in the reagents to avoid potential underestimation. 75 On the other hand, this can also be considered a disadvantage since such assays with added AT measure the concentration of heparin irrespective of whether the patient has sufficient AT for the heparin to exert its full protective action in vivo. 75 The availability of anti-Xa assays may be less than the availability of aPTT in some countries and the test cost is usually greater than aPTT. Overall, there are fewer disadvantages related to anti-Xa compared to aPTT for monitoring UFH. The authors believe that anti-Xa is the better option, but it should be noted that there is no strong evidence based on clinical outcome data to support that anti-Xa is superior to aPTT in monitoring UFH. When anti-Xa is used (for UFH monitoring) the assay should be calibrated using a UFH calibrator traceable to the current international standard for UFH. 18

## Monitoring of Unfractionated Heparin during Cardiopulmonary Bypass

As described above, UFH is the standard anticoagulant used for CPB due to extensive experience in its use for this procedure, proven clinical efficacy and safety, rapid onset of action, ability to neutralize rapidly by protamine sulfate, and low cost. 83 High-dose UFH, generally 300 to 400 U/kg, is given intravenously during the CPB. 83 Activated clotting time (ACT) is a point-of-care test (POCT) using whole blood to monitor UFH in this setting and generally it is aimed to achieve an ACT >480 seconds during the CPB. 83 Since ACT is a POCT, it is easy to use, and the anesthetic team monitors the adequacy of heparinization by checking the ACT every 30 to 40 minutes. 83 However, ACT is affected by various other factors in addition to heparin, including activator used (i.e., celite, kaolin, or glass beads), 84 hypothermia, platelet count, and hemodilution. 85 Therefore, it is not surprising that ACT has poor correlation with the plasma heparin level 83 and is a poor predictor of bleeding risk. Furthermore, ACT may not accurately predict the protamine sulfate dose needed for reversal following the completion of the CPB. 86 These effects are even more prominent in patients with LA in whom baseline ACT is affected by the presence of LA. 87

We previously assessed the correlation between ACT and heparin anti-Xa level in a single-center cohort study of 19 patients with LA with or without APS undergoing CPB. It was found that the correlation between heparin anti-Xa level and the ACT was poor (r = 0.16, *p* = 0.46). The median ACT and heparin anti-Xa level at the end of CPB prior to protamine sulfate administration were 630 seconds (540–910) and 4.5 IU/mL (range: 4.0–6.2), respectively. 87 None of the patients had significant bleeding requiring transfusion, or thrombosis within 30 days. 87 Although monitoring the inhibition of FXa by heparin is considered the gold standard for measuring the anticoagulant effect of UFH, many hospital laboratories are not able to provide the heparin activity, as measured by the anti-Xa level, in a timely manner for patients undergoing CPB, making it challenging to use in routine clinical practice. 87

A heparin anti-Xa level >4.0 IU/mL is required during CPB. 85 However, the standard heparin anti-Xa assays become nonlinear at levels above 1.5 to 2.0 IU/mL, and some manufacturer(s) instructions prohibit diluting the specimen. Nevertheless, serial dilutions of the sample in pooled normal plasma are required to obtain accurate levels above 2 IU/mL. 87 A point-of-care device is available to estimate the free plasma heparin level in whole blood (The Hepcon Hemostasis Management System [Hepcon HMS; Medtronic, Minneapolis, MN]), 88 89 which is shown to correlate well with heparin anti-Xa during CPB in some studies. 90 91 92 However, the role of this device in patients with LA has not been evaluated.

Although our institution's approach is monitoring the heparin anti-Xa levels in patients with LA undergoing CPB, the practice may vary widely depending on the availability of resources. The use of ACT with no change in the standard ACT goals for CPB (usually 400–480 s) is one of them. If the baseline ACT is not affected by the presence of LA or is increased by only few seconds, this approach is reasonable. Maintaining an ACT level that is twice the normal range, adjusting ACT range for CPB based on baseline ACT, or creating a patient-specific ACT titration curve is also used in clinical practice. 87

## Conclusion

Heparin has pleotropic effects beyond its anticoagulant effect, with anti-inflammatory, anticomplement, and antiproliferative activities. In certain situations, such as obstetric APS, the nonanticoagulant effect may be more important, especially for prevention of recurrent early miscarriage. Nonanticoagulant effects of heparin may need further research, with the aim to develop potentially other therapeutic agents expressing similar nonanticoagulant properties to UFH for use in inflammatory disorders. Although, there is no randomized, double-blind, controlled studies comparing aPTT versus anti-Xa or ACT in monitoring of UFH, evidence suggests that there is a poor correlation between aPTT and heparin anti-Xa and ACT and heparin anti-Xa levels. 10 83 87 Laboratories need to establish their local aPTT UFH therapeutic reference interval for treatment. The poor correlation is more marked during acute illness (for instance, patients in intensive care units), presence of LA, and during pregnancy. Use of a heparin anti-Xa assay is preferable to ACT for the measurement of heparin effect to avoid circuit thrombosis and to judge correct protamine sulfate dosing for reversal, especially in patients with LA undergoing CPB. Overall, heparin anti-Xa may be a better indicator of anticoagulant effect of UFH than aPTT due to various confounding effects of aPTT. Larger studies assessing the clinical outcomes and transfusion requirements based on aPTT versus heparin anti-Xa to assess anticoagulant effects of UFH are required.
